# The Mechanism of Proinflammatory HDL Generation in Sickle Cell Disease Is Linked to Cell-Free Hemoglobin via Haptoglobin

**DOI:** 10.1371/journal.pone.0164264

**Published:** 2016-10-07

**Authors:** Xiang Ji, Yimin Feng, Hui Tian, Wei Meng, Weiling Wang, Na Liu, Jun Zhang, Lingshu Wang, Jian Wang, Haiqing Gao

**Affiliations:** 1 Geriatric Department Qilu Hospital of Shandong Univeristy; Shandong Key Laboratory of Proteomics, Jinan 250012, Shandong, China; 2 Clinical Laboratory, Qilu Hospital of Shandong Univeristy, Jinan 250012, Shandong, China; 3 ICU, The Affiliated Hospital of Qiingdao University, Qingdao 266012, Shandong, China; 4 Cardiology Department, Qilu Hospital of Shandong Univeristy, Jinan 250012, Shandong, China; 5 Pharmacological Laboratory, Qilu Hospital of Shandong Univeristy, Jinan 250012, Shandong, China; 6 Endocrinology Department, Qilu Hospital of Shandong Univeristy, Jinan 250012, Shandong, China; Heart Research Institute, AUSTRALIA

## Abstract

In sickle cell disease (SCD), the inflammatory properties of high-density lipoprotein (HDL) can be changed by cell-free hemoglobin (Hb), which is released into the blood during hemolysis. Hb in the plasma of SCD patients or mice can bind with HDL specifically inducing an inflammatory reaction. In our study, we found increased amounts of inflammatory factor proteins in the chronic oxidative state of SCD with higher levels of Hb, haptoglobin (Hp) and hemopexin (Hx) in the apolipoprotein A-I (ApoA-1) particles of HDL and the role of HDL is changed from being anti-inflammatory to proinflammatory. Our results also suggest Hp and Hx, the scavengers of Hb in HDL, are positively associated with inflammatory levels in SCD patients. HDL retained its inflammatory inhibition role in Hp−/− mice, with less Hb accumulation. Hx may further prevent inflammatory reaction because its level will be even higher when lack of Hx. We therefore demonstrated that Hp is indispensable during the process whereby Hb associates with HDL and plays a clear proinflammatory role. Therefore, it is essential to break the binding between Hb and Hp for treatment. The dissociation of Hb/Hp/Hx complexes may also play an important role in the study of other inflammatory angiogenesis-related diseases.

## Background

In 2016, American College of Cardiology (ACC) revealed the results of the ACCELERATE study and demonstrated once again that there was no benefit for patients if only HDLc can be increased by using a CETP inhibitor [1]. Among the explanations given by experts, one question tthat was raised was “Should HDL particles be the ‘good cholesterol’?”. Surprisingly, the answer might be ‘not always’ as HDL may be changed into a pro-inflammatory lipoprotein during an inflammatory response within the body. Sickle cell disease (SCD) is a form of hemoglobinopathy that is characterized by chronic hemolytic anemia, followed by a remarkable inflammatory response and oxidative stress within the circulation. Damaged sickle cells can release cell-free hemoglobin (cf-Hb) into the circulation, which induces hemoglobinuria, hypertension and pulmonary hypertension, along with other pathophysiological vascular changes. The symptoms of SCD begin in early childhood and last throughout life [2, 3]. However, the symptoms of SCD are closely related to vascular inflammation caused by abnormal HDL. Inflammation might be a more important factor than lipid infiltration for vascular lesions, even in atherosclerosis [4].

We have also reported that lower total cholesterol levels were found in the plasma of most people with SCD[5]. Recent studies have however demonstrated that the inflammatory properties of HDL impact the oxidative levels of SCD patients and animal models more significantly and with greater influence than does the amount of HDL [6, 7]. Healthy HDL can inhibit low-density lipoprotein (LDL) oxidation and other inflammatory complex formation. However, damaged HDL has the opposite effect and is considered to be a proinflammatory factor [8].

Although less commonly recognized, proinflammatory HDL (p-HDL) remains a good marker, emphasizing the role of HDL in the inflammatory process, in addition to the quantity of HDL [5]. Moreover, p-HDL contains lower levels of anti-inflammatory/oxidant proteins, such as apolipoprotein A-I (ApoA-I) and paraoxonase 1 (PON1) [9]. Effluxed cholesterol subsequently becomes reduced without sufficient ApoA-1 [10]. On the other hand, increased levels of heme, and other proinflammatory factors, act as an oxidant challenge to the HDL cargo. HDL therefore not only impairs LDL oxidative inhibition, but also promotes more serious inflammation. In previous study, our laboratory made a new and more accurate method to quantify p-HDL in the plasma of SCD patients using an “Anion-Exchange HPLC-Post Column Reaction” system (AE-HPLC-PCR). However, the molecular mechanisms that convert anti-inflammatory HDL into a proinflammatory HDL are not yet well understood.

It has been well demonstrated that increased cf-Hb in SCD plasma is one of the most important reasons for this conversion to p-HDL. Large quantities of pro-oxidative cf-Hb and heme are released by sickle cells during hemolysis in SCD. We have measured cf-Hb in plasma and ApoA-I particles in SCD samples and discovered that the concentration of cf-Hb was increased and directly promoted p-HDL levels [11]. Based on these points, Hb is a new target for study and treatment to decrease p-HDL and prevent inflammation and oxidative reactions in SCD. Therefore, this study was conducted to explore the molecular mechanisms involved in the association of Hb and HDL.

The oxidative injury from Hb is enhanced with the presence of heme. As a toxic factor with oxidative properties, heme can lead to tissue injury by taking part in the process of reactive oxygen species (ROS) production (the Fenton reaction) [12].

It has also been reported that haptoglobin (Hp) and hemopexin (Hx) have a very powerful affinity to bind Hb. Hp and Hx are part of the family of acute phase proteins and are considered to be Hb scavenger proteins [13]. They are mainly produced in the liver and their synthesis is induced by an inflammatory environment [14]. Large quantities of Hb can be released into the circulation during hemolysis causing a very high cell-free Hb level in plasma. Meanwhile, the scavenger proteins, Hp and Hx, have the ability to prevent cf-Hb causing oxidative toxicity [2,15]. Normally in humans or animals, there is only a low level of cf-Hb, but abnormally raised levels of cf-Hb in SCD can associate with HDL and change HDL into p-HDL. Normally Hb can mostly be found in non-lipoproteins [10]. The metabolism of Hb is completed by macrophages that internalize and degrade Hb or Hb–Hp complexes through the scavenger receptor CD163 [16]. Hx–heme is removed by the LDL-receptor-related protein CD91 through receptor-mediated endocytosis, suggesting a close relationship between Hb, Hp and Hx [17].

These studies indicate that the inflammatory processes involving Hb may also involve Hp and Hx and their related signaling pathways. We therefore wished to discover whether Hp and Hx also participated in the binding between Hb and HDL, and what their influence was on the proinflmmatory effect of HDL.

## Method and Materials

### Human subjects

All of the patients included in this study had SCD and were recruited at Qilu Hospital of Shandong University from June to December, 2015. In all cases, approval was given for the total of 60 plasma samples, whether from patients or healthy donors, to be used in this study before the samples were obtained. Informed verbal consent was obtained from control subjects and patients or healthy subjects. The human subjects were well-organized in formal recruitment, including ethical consent, information acquisition, and the collection of abandoned blood samples after clinical blood tests. Institutional Review Board approval was obtained from the Medical Ethics Committee of Qilu Hospital of Shandong University, which was obtained for studies involving samples from human subjects in accordance with Declaration of Helsinki and its revised version in 2000. An ulnar vein puncture was used to obtain the blood samples. All investigators were blinded to the participant group and did not have access to the identity of the groups before the data collection and statistical analysis were completed.

### Animal experiments

All animal experiments were approved by the Institutional Animal Care and Use Committee (IACUC) of Shandong University. Plasma was pooled from C57BL/6J, Hp−/−, Hx−/−, SCD female mice between the ages of 8 and 12 week (eight mice per group). For the D-4F treatment, SCD mice were intraperitoneally injected with D-4F (10 μg/day for four weeks) at the age of eight weeks. This study was carried out in strict accordance with the recommendations in the Guide for the Care and Use of Laboratory Animals of the National Institutes of Health. All surgical protocols used to obtain the plasma from the inferior vena cava were performed with the animals under complete ketamine and xylazine anesthesia by intraperitoneal injection and all efforts were made to minimize suffering and pain.

### Lipid levels in plasma

Lipid measurements in human or mice plasma included total cholesterol, HDL cholesterol (HDLc) and LDL cholesterol (LDLc). All three types of cholesterol were quantitatively measured using an enzyme reactive kit (Wako, Osaka, Japan).

For HDLc data, HDL was separated from plasma by precipitation of ApoB-containing lipoproteins as follows: 20mg Dextran sulfate (Dextran sulfate 50, Warnick & Co., Arizona, USA) was dissolved in 1mL deionized water to be a final concentration of 20 mg/mL, then was mixed with 1M MgCl_2_ at the ratio of 1:1. 60 μL plasma was combined with 6 μL of the mixed reagent. After 10 min, the precipitate was removed to leave the supernatant for testing.

### Plasma p-HDL level and the detection of ROS in plasma

p-HDL levels and ROS in plasma were quantified by spectrometry with 2,7’-dicholrofluorescein diacetate (DCFH; Calbiochem, California, USA). HDL was isolated and collected by AE-HPLC (Agilent 1100) as previously described [5]. The sample was concentrated by a centrifugal filter unit (Regenerated Cellulose 3000 MWCO, Amicon Ultra, Billerica, Massachusetts, USA) and HDLc was measured as above. A 1μg sample from patients or healthy donors was used to assess oxidation by plate assay as previously described [5, 18]. The p-HDL level was obtained from the linear slope by calculation.

A dichlorofluorescin (DCFH) assay is commonly used to monitor the levels of reactive oxygen species (ROS) by using 2’,7’-Dichlorofluorescin diacetate (DCFH; Sigma-Aldrich, Missouri, USA). The wavelength used to detect the fluorescence was Ex488 nm and Em525 nm. Reactive nitrogen species (RNS) measurement was conducted with 4-Amino-5-methylamino-2',7'-difluorofluorescein diacetate (DAF-FM diacetate; Sigma-Aldrich, Missouri, USA). The wavelength used to detect fluorescence was Ex485nm, Em530nm.

### Lipoprotein separation by HPLC

AE-HPLC (Agilent 1100) was used to separate lipoprotein from plasma as previously described [5]. The principle of AE-HPLC is the elution of five types of lipoprotein from an anion exchange column (Tosoh, Tokyo, Japan) using different percentages of elution buffer, which was altered by changing the percentage B Buffer. Eluent A was 50mM Tris-HCl, 1mM (EDTA-2Na), 2H2O; and 500 mM NaClO4 was added into Eluent A to create Eluent B. To isolate HDL, the ionic strength of the elution buffer was altered because the percent of the B Buffer can be changed in the program of HPLC software. Then, 18% Eluent B was held for 15 minutes to spearate the HDL. This system was connected with a post-column reactor system that reacted lipoprotein with DCFH and oxidants to produce p-HDL data [5]. It has a fluorescence detector to measure the fluorescence from DCFH (Ex485nm, Em530nm).

### The Association between HDL and hemoglobin, haptoglobin, hemopexin

We used immunoprecipitation to obtain HDL/ApoA-1 particles from the plasma. Anti-ApoA-I (Goat; Rockland, Massachusetts, USA) and anti-HDL (Abcam, Massachusetts, USA) were labeled with biotin (1:1) to bind with streptavidin beads. The protein particles eluted from beads were then obtained for immunoblot analysis as previously described [19]. The first antibodies used (all Santa Cruz, California, USA) were as follows: anti-ApoA-I (1:2000), anti-hemoglobin (1:1500), anti-haptoglobin (1:2000) and anti-hemopexin (1:1000).

We also sought to determine the association between these relative proteins using Bio-Layer Interferometry (BLI) by Octet RED station (FB-10206; Forte Bio). This machine was used to measure the kinetics of protein-protein interactions [20]. We labeled 1μg particles from plasma or hemoglobin antibody (Meridian, Tennessee, USA) with Biotin (1:1). The concentration of antibody was 1μg/ml. The principle of Octet is that it can quantify proteins (antigen or antibody) that are caught directly or indirectly by sensors in this system. The Hb/Hp/Hx antibody was labeled with biotin for attachment to a sensor. First, binding occurs between the specific protein’s antibody and the sensor, which is achieved by biotin–streptavidin. Then, once the sample has been loaded (e.g., HDL/ApoA-1 particles) the sensors are forced to make contact with the sample. Finally, we were able to further detect what protein had bound to the particles via specific antigen-antibody binding. The effective and strong association among proteins (e.g., associated Hb and Hp on HDL/apoA-1) cannot be removed by phosphate-buffered saline (PBS) washing for 200 s, and is shown by upward curves.

### Inflammatory factor measurement

As well as measuring the generation of ROS, the arylesterase activity of PON1 in HDL was measured using phenyl acetate as the substrate as previously described [18]. An aliquot of 20μl HDL was mixed with 100 mM phenyl acetate and 100mM CaCl_2_ in 40mM Tris-HCl buffer (pH8.0) for 6 minutes at 25°C. Initial rates of hydrolysis were determined at a wavelength of 270nm on a spectrophotometer (Beckman Coulter^®^ Instruments, Brea, California, USA), with 1unit of arylesterase activity being equivalent to 1mM phenyl acetate hydrolyzed in 1L every minute.

The lipid hydroperoxide (LOOH) level was determined by lipid hydroperoxide assay kit (Cayman, Michigan, USA) with the absorbance at 500nm being read in a microplate reader. This assay can measure the hydroperoxides directly by their redox reactions with ferrous ions.

Monocyte chemotaxis activity (MCA) was assessed by quantifying the number of monocytes that migrated into six standard high-power fields by MCP-1 as previous reported [21, 22]. The HDL inflammatory index (HII) was calculated based on the MCA data: p-HDL results in a value >1.0, whereas it is anti-inflammatory if the value is <1.0.

### ELISA assays for Hb, Hp, Hx, hs-CRP, IL-6, IL-10, plasma heme /HDL

Hb, Hp, Hx, high-sensitivity C-reactive protein (hs-CRP), interleukin 6 (IL-6), and IL-10 were quantified by following the protocol of enzyme-linked immunosorbent assay (ELISA) kits (TSZ ELISA, Oregon, USA). All of the samples were incubated in 96-well assay plates for 1 hour. Diluted specific primary antibodies for the kit were added into the wells and left for 2 hours. The primary antibodies were detected using a horseradish peroxidase (HRP)-conjugated secondary antibody for 30 minutes. This was mixed with chromogen A and B, and the colorimetric reagent was added into wells for 30 minutes, following which stop buffer was added and the data were read at OD450. A standard curve was created to convert the OD450 of each sample to the concentration of measured protein.

Heme in plasma was determined using the appropriate assay kit (Abnova, California, USA) using the principle of improved aqueous alkaline solution method.

### Proteins associated with ApoA-1 in HDL

Sandwich ELISA was used to detect Hb, Hp and Hx in HDL containing ApoA-1. To a 96-well ELISA plate were added 5μg/ml Anti-ApoA-1 (Goat; Meridian, Massachusetts, USA) and anti-HDL (Abcam) and the plate was then incubated overnight at 4°C. The wells were then loaded with an aliquot of HDL or plasma for 1 hour. They were then incubated with the primary antibodies at their respective dilution ratios of 1:2000 for all mouse antibodies, 1:2000 for human Hb and 1:2500 for human Hp, Hx and ApoA-1 antibodies. Further incubations were performed for HRP-conjugated secondary antibodies and the chromogen reagent as per the protocol for the ELISA kit (R&D Systems, Nebraska, USA). The data was read with OD450 and calculations were performed similarly to the previously stated method.

### Statistical analysis

Data are presented as mean ± standard deviation (SD) unless otherwise indicated. Correlations were made by linear regression. The statistical calculation and analysis were performed using Graph Pad Prism 5.0 software. Comparisons between two groups were made by a t-test. Comparisons between multiple groups were made by one-way ANOVA with the Bonferroni correction for multiple groups. In figures, “relative” represents the average of the concentration relative to wild type (WT; control). Minimum levels of significance were set at p<0.05. Relevant calculations and statistical analysis were performed using SPSS 17.0.

## Results

### Lipid profiles of SCD patients and healthy donors

Plasma samples from 32 SCD patients were used in this study. All of the samples were obtained legally. Plasma samples from 28 healthy donors were used as controls (Table 1). There were a total of 18 females in the SCD group and 15 females in the control group. Mean age: 40.2 years ± 7.5 years (SCD) vs. 38.2 years ± 6.2 years (Control). It can be seen in this table that the total cholesterol and HDLc levels were lower in those with SCD than in the control group (Table 1).

**Table 1:** Lipid profiles of the plasma from 32 sickle cell disease (SCD) patients and 28 healthy normal donors.

	Healthy donors(Mean ± SD)	SCD(Mean ± SD)	p*
Total Cho (mg/dL)	135.7±33.1	103.9±27.6	< 0.05
HDL (mg/dL)	58.1± 17.2	31.3± 10.9	< 0.05
LDL (md/dL)	74.6 ± 28.7	71.0 ± 38.6 NS	

SD, standard deviation; HDL, high-density lipoprotein; LDL, low-density lipoprotein; NS, not significant.

* analyzed by a *t-test*.

### Inflammatory levels in plasma

The inflammatory factors hs-CRP, heme, ROS, RNS, IL-6, IL-10 and p-HDL were measured to show the total inflammatory level in plasma. It can be seen that the inflammatory markers in SCD patients were much higher than those in the healthy donors with significant differences being seen (Table 2).

**Table 2:** Levels of inflammatory markers in plasma in 32 sickle cell disease (SCD) patients and 28 healthy normal donors.

	Healthy donors	SCD	p*
	(Mean ± SD)	(Mean ± SD)	
hs-CRP (mg/L)	5.42±0.89	78.63±13.20	< 0.01
PON1(U/mL)	127.4± 38.2	236.6±49.1	< 0.01
Heme(mM)	4.1±0.6	13.3±1.8	< 0.001
ROS (RFU)	162.1±70.4	353.2±81.9	< 0.001
RNS (RFU)	56.8±4.3	68.3 ± 5.2	< 0.05
p-HDL (RFU/μg HDLc·min)	8.84±2.05	23.76±4.81	< 0.001
IL-6 (Relative concentration)	1.00±0.36	1.77±0.84	< 0.001
IL-10 (Relative concentration)	1.00±0.28	1.31±0.66	< 0.01

hs-CRP, high-sensitivity C-reactive protein; PON1, paraoxonase 1; ROS, reactive oxygen species; RNS, reactive nitrogen species; p-HDL, Proinflammatory high-density lipoprotein; IL, interleukin; RFU, relative fluorescence unit.

* analyzed by *t-test*.

### Hb, Hp and Hx levels in plasma and HDL

Plasma Hb, Hp and Hx protein levels were measured by ELISA assays. Hb, Hp and Hx were higher in the SCD patients compared with healthy donors. However, ApoA-1 was reduced in the plasma or HDL (Table 3A).

**Table 3:** Levels of hemoglobin (Hb), haptoglobin (Hp) and hemopexin (Hx) associated with HDL in 32 sickle cell disease (SCD) patients and 28 healthy normal donors (Control).

A			
ELISA (Relative concentration)	Control(Mean ± SD)	SCD(Mean ± SD)	p*
Hemoglobin	1.00±0.43	3.74±0.31	< 0.001
Haptoglobin	1.00±0.45	1.87±0.68	< 0.001
Hemopexin	1.00±0.34	1.54±0.52	< 0.001
ApoA-1	1.00±0.49	0.72±0.26	< 0.05
B			
HDL-captured ELISA	Control(Mean ± SD)	SCD(Mean ± SD)	p*
(Relative concentration)			
Hemoglobin	1.00±0.35	2.58±1.24	< 0.0001
Haptoglobin	1.00±0.32	2.14±0.79	< 0.0001
Hemopexin	1.00±0.41	1.41±0.51	< 0.05
ApoA-1	1.00±0.32	0.78±0.42	<0.05
C			
ApoA-1-captured ELISA	Control(Mean ± SD)	SCD(Mean ± SD)	p*
(Relative concentration)			
Hemoglobin	1.00±0.43	3.02±1.68	< 0.0001
Haptoglobin	1.00±0.38	2.31±0.97	< 0.0001
Hemopexin	1.00±0.21	1.53±0.38	< 0.01
ApoA-1	1.00±0.27	0.97±0.30	NS

ELISA, enzyme-linked immunosorbent assay; SD, standard deviation.

*analyzed by a *t-test*.

To show any differences in the Hb/Hp/Hx complexes associated with ApoA-1 in HDL, we also performed a sandwich ELISA to capture ApoA-1 particles and found that Hb/Hp/Hx complexes were increased in the ApoA-1 particles from HDL (Table 3B and 3C).

### Relationship between lipid hydroperoxide/HDL inflammatory index and Hb/Hp/Hx/ApoA-1

**Fig 1:**
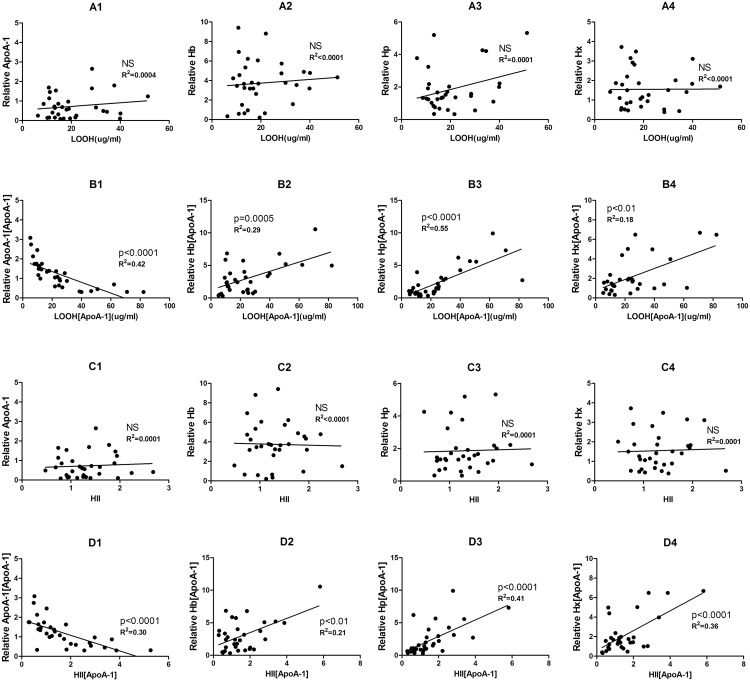
Plasma hemoglobin (Hb), haptoglobin (Hp) and hemopexin (Hx) in HDL are linearly correlated with lipid hydroperoxide (LOOH) and HDL inflammatory index (HII). “[ApoA-1]” means “in ApoA-1 particles”. Results in SCD patients (n = 32): ApoA-1, Hb, Hp and Hx levels in plasma (A1–-A4, respectively) or ApoA-1 particles, Hb, Hp, and Hx associated with ApoA-1 (B1–B4, respectively) vs. LOOH content in ApoA-1 particles. ApoA-1, Hb, Hp and Hx levels in plasma (C1–C4) or those associated with ApoA-1 (D1–D4) vs. HII measured by monocyte chemotaxis activity (MCA). Linear regression was performed individually and p values and R^2^ values are shown in the figures. (NS = not significant).

p-HDL is characterized by an HII >1.0, which is considered an important indicator of the inflammatory properties of HDL, along with the accumulation of LOOH, oxidizable phospholipids and other inflammatory factors. To determine whether Hb, Hp, Hx are related to these inflammatory indicators, plots of the plasma levels of Hb Hp Hx and ApoA-1 particles of HDL were correlated with LOOH and HII, respectively (p< 0.05) (Fig 1). It can be seen that there is a linear correlation only in SCD patients (only the data with statistical significance are shown). ApoA-1 is negatively linked with inflammatory levels of HDL (Fig 1B1 and 1D1). The calculation for the regression analysis mentioned above was conducted using the SPSS Satistics 17.0 software package. Statistical analysis of the data reveals that the cf-Hb that associates with ApoA-1 holds a positive relationship with LOOH and HII. In addition, Hp and Hx in ApoA-1 particles show similar correlations with these two markers (Fig 1B2–1B4 and 1D2–1D4). Linear regression indicates a positive relationship between increased cf-Hb in HDL and the accumulation of LOOH and HDL proinflammatory properties in SCD patients. It is also possible to see the secondary increase in its scavenger proteins, Hp and Hx. We have however not found any similar links in the group of healthy donors (S1 Fig).

### Hb levels in ApoA-1 particles are positively correlated with heme, ROS, and IL-6/IL-10 levels

**Fig 2:**
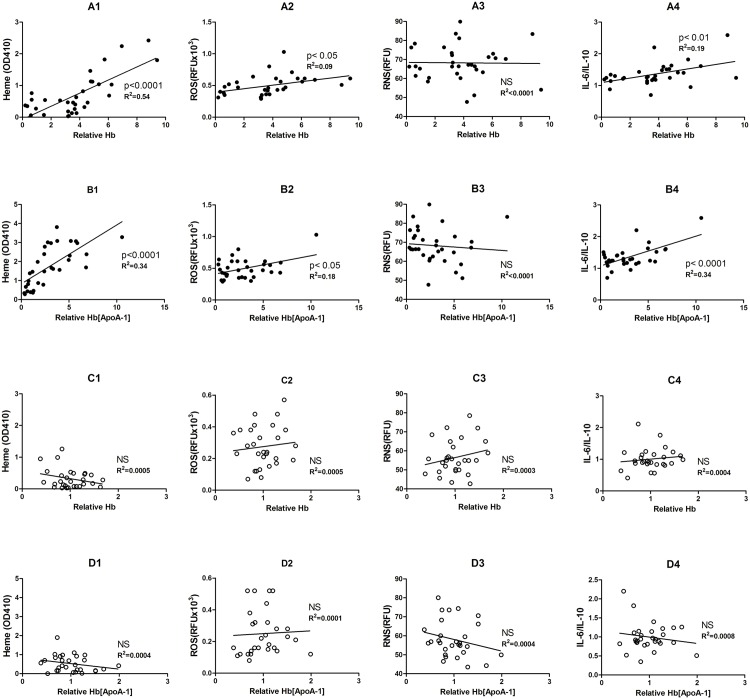
Hemoglobin (Hb) concentrations associated with HDL were positively correlated with heme, reactive oxygen species (ROS) level and IL-6/IL-10. Relative Hb levels in the plasma (A1–A4 and C1–C4). Relative Hb [ApoA-1] = Hb associated with ApoA-1 particles from HDL (B1–B4 and D1–D4). Fig A1-A4 and B1-B4 present the data from the SCD group (n = 32) and Fig C1-C4 and D1-D4 are those from healthy donors (n = 28). Linear regression was performed individually and and p values and R^2^ values are shown in the Figs. (NS = not significant).

The relationship between cf-Hb and p-HDL has been demonstrated previously [5]. In this study, we have again shown correlations in the plots of cf-Hb in ApoA-1 with the factors heme, ROS, RNS, and IL-6/IL-10 that reflect the systemic inflammatory levels in plasma. Linear regression shows that the concentration cf-Hb in ApoA-1 is positively linked with the heme, ROS and IL-6/IL-10 levels in SCD (Fig 2A and 2B). However, the same linear correlation does not exist in the plasma of healthy donors (Fig 2C and 2D).

### The detection of the levels of Hb, Hp, Hx associated in HDL

**Fig 3:**
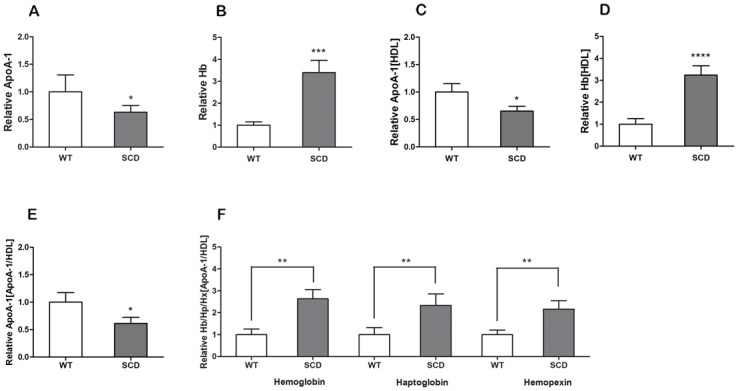
Hemoglobin (Hb), haptoglobin (Hp) and hemopexin (Hx) content is increased in ApoA-1 particles of HDL in SCD mice vs. wild-type (WT) mice. (A,B) ApoA-1 and Hb levels, respectively, in the plasma of SCD mice (n = 8) and WT mice (n = 8). (C,D) ApoA-1 and Hb levels, respectively, in HDL measured by sandwich ELISA. (E) Immunoprecipitation was used to obtain the ApoA-1 particles. We measured the level of these particles by ELISA and found fewer ApoA-1 particles in the HDL of SCD mice compared to WT. (F) Hb, Hp and Hx levels associated with ApoA-1 of HDL in the two groups. The association of ApoA-1 with Hb/Hp/Hx was enhanced because ApoA-1 was reduced in the HDL of the SCD sample.

**Fig 4:**
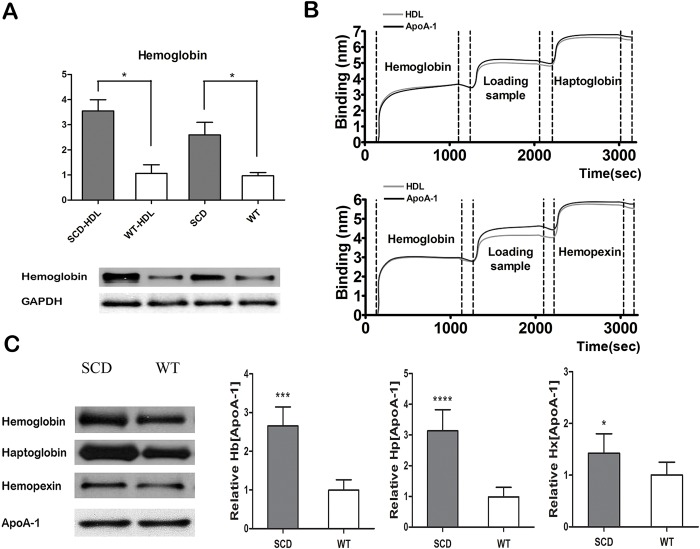
The association of Hb/Hp/Hx complexes with HDL in SCD mice. (A) Cell-free hemoglobin (cf-Hb) or associated Hb was elevated in the HDL or plasma of SCD mice compared to wild-type (WT). (B) The curve in the Octet system shows HDL had its association for Hp (upper) and Hx (lower) on the basis of HDL/ApoA-1 inside HDL. Hemoglobin/Haptoglobin/ Hemopexin = loading biotin-labelled Antibody of Hemoglobin/Haptoglobin/Hemopexin. It suggests that Hb/Hp/Hx association has a strong relationship with ApoA-1 particles in HDL. (C) The result of immunoprecipitation and immunoblot analysis show that Hb, Hp and Hx associated with ApoA-1 was increased under conditions in which ApoA-1 was at the same level in both groups. * p<0.05; *** p<0.001; **** p<0.0001.

Direct and indirect (sandwich) ELISAs were performed to measure the amounts of Hb, Hp, Hx and ApoA-I. Fig 3A, 3C and 3E shows the ApoA-1 level was decreased in plasma and HDL of SCD mice. However, cf-Hb released by sickled red cells showed the opposite result (Fig 3B and 3D). We also observed significant increases in Hb, Hp and Hx in ApoA-1 particles of HDL in SCD mice (Fig 3F). Based on linear regression and the data above, it appears that ApoA-I is necessary for the combination between HDL and Hb/Hp/Hx complexes. Furthermore, we demonstrated by the immuoprecipitation and immunoblot methods that the amount of Hb associated with HDL was much higher than in the controls (Fig 4A).

In our study, we used Octet Red to quantify the interaction between proteins and compare the affinity among protein groups. According to the curve in Fig 4B, HDL cargo and ApoA-1 particles exhibit similar binding curves with Hb, Hp, Hx without removal by washing. The effective affinity among these proteins and Hb/Hp/Hx complexes that associate with HDL is confirmed as being through ApoA-1 particles. The result of the immunoprecipitation/immunoblot also reveals the high affinity of the ApoA-1 and Hb/Hp/Hx complexes (Fig 4C).

### The role of Hp in the mechanism of the association between Hb and HDL

**Fig 5:**
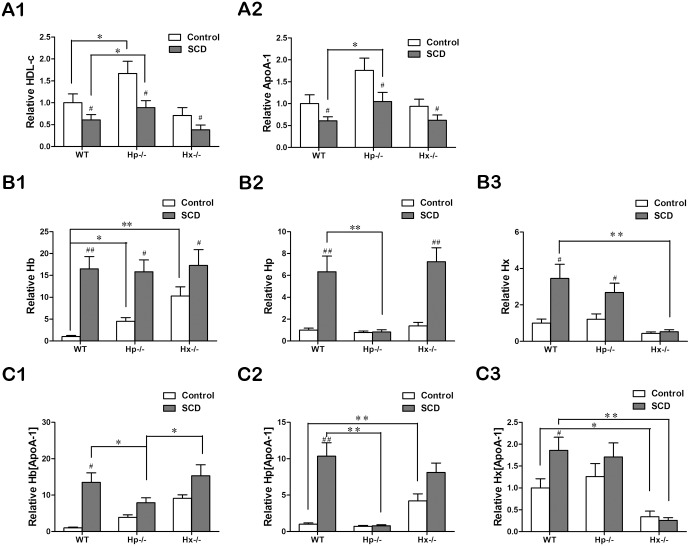
Hemopexin (Hp) is the protein necessary to promote the association of hemoglobin (Hb) with HDL. (A) HDL cholesterol (A1) and associated ApoA-1 (A2) concentrations in C57BL/6J wild-type (WT), Hp−/− or Hx−/− mice with or without SCD (n = 8 each). (B,C) Hb, Hp and hemopexin (Hx) in plasma (B1–B3, respectively), determined by direct ELISA, and in ApoA-1 (C1–C3, respectively), determined by sandwich ELISA. *ANOVA* were performed for statistical analysis. * = p<0.05, ** = p<0.01; # = p<0.05, ## = p<0.01. The symbol “#” shown statistical difference between SCD and control in same genotype.

To determine the role of Hp and Hx in the association between Hb and HDL, we performed research to determine whether they are necessary for this process using Hp and Hx knock-out in WT and SCD mice (Table 4). HDLc levels are shown in Fig 5A1. HDLc levels in Hp−/− non-SCD and SCD mice were higher than their counterparts in WT group. ApoA-1 levels of HDL were measured by ELISA (Fig 5A2), and ApoA-1 were decreased in the SCD group, compared with their counterparts in the non-SCD group. For the SCD types, ApoA-1 of Hp−/− mice was higher than in the others. The levels of Hp and Hx confirmed the effective knock-out as Hp and Hx can be found in much lower levels of Hp−/− and Hx−/− mice (Fig 5B2 and 5B3). Hp−/− mice held weakly associated Hb in the HDL, but not with cf-Hb in plasma. And cf-Hb was increased in Hp−/− and Hx−/− mice of Control group, compared with WT (Fig 5B1 and 5C1). Similarly decreased Hb combined with HDL was not observed in SCD of Hx−/− mice. We therefore concluded that Hp is the necessary protein in the mechanism for the association between Hb and HDL. Furthermore, we found that ApoA-1-associated Hp in both two sorts of Hp−/− mice was significantly lower than in the WT mice (Fig 5C2). It should be noted that total Hp shows an ascending tendency in Hx−/− mice with SCD (Fig 5B2). ApoA-1-associated Hp was found at an even higher level in Hx−/− mice without SCD than in the WT mice (Fig 5C2).

**Table 4:** Plasma lipoprotein levels (mg/dL) in wild-type (WT), Hp−/− and Hx−/− mice with and without sickle cell disease (SCD; n = 8 each group).

	Control			SCD		
	WT	Hp-/-	Hx-/-	WT	Hp-/-	Hx-/-
HDL	51.3±7.3	87.9±7.6*	36.7±5.7*	30.3±3.3	37.6±6.1	17.4±3.3^#^
LDL	67.1±10.3	59.2±10.4*	70.8±14.2	81.2±14.0	98.1±12.1^#^	65.8±11.9^#^

HDL, high-density lipoprotein; LDL, low-density lipoprotein; Hp, haptoglobin; Hx, hemopexin; SD, standard deviation.

(* = p<0.05 by t-test compared with WT in control group, # = p<0.05 by a *t-test* compared with WT in SCD).

### The role of Hp in p-HDL

**Fig 6:**
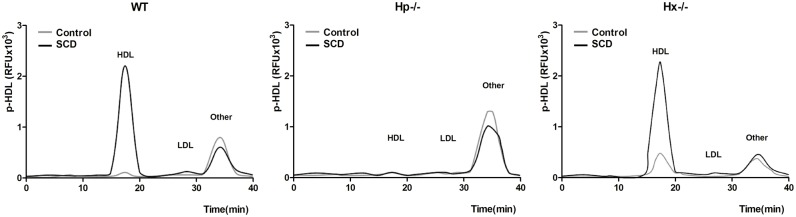
Hemopexin (Hp) is required to increase levels of p-HDL in SCD. The lipoprotein fractions were isolated by HPLC assay to detect p-HDL (accumulation of ROS) in wild-type (WT), Hp−/− and Hx−/− mice. A significantly lower level of p-HDL is seen in SCD mice with Hp knock-out, compared with other SCD groups, confirming that Hp is the protein necessary for the proinflammatory properties of HDL. (Other = other fractions of lipoprotein; RFU, relative fluorescence units).

The production of ROS in the lipoprotein fractions in plasma was determined by HPLC. An accumulation of p-HDL was found in SCD (WT and Hx−/−), but levels were significantly reduced in Hp−/− mice (Fig 6). This shows that the presence of Hp promotes the accumulation of p-HDL in lipoprotein fractions.

**Fig 7:**
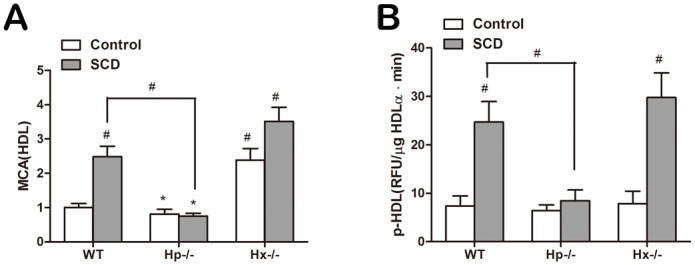
Hb/Hp/Hx complexes play a role in the proinflammatory functions of HDL. HDL was separated from wild-type (WT), Hp−/− and Hx−/− mice either with or without SCD (n = 8 each). (A) The HDL inflammatory index (HII) was measured by monocyte chemotaxis activity (MCA). (B) Proinflammatory HDL (p-HDL) level was measured by plate assay after HDL was isolated by HPLC as previously described. HII<1 and lower p-HDL level were only found in the Hp knock-out SCD mice. Comparisons were performed by *ANOVA* (* = p<0.05; # = p<0.01).

To further determine the role of Hp and Hx in the inflammatory properties of HDL, we assessed the HII of all six types of mice by MCA to understand their inflammatory properties (Fig 7). A large amount of HDL became proinflammatory in SCD without knock-out of Hp. In Hp−/− mice, HDL was both pro-/anti-inflammatory even with SCD. Meanwhile, p-HDL was found in all Hx−/− mice, which in part demonstrated the anti-inflammatory properties of Hx (Fig 7A). The plasma p-HDL level in Hp−/− mice was not as high as in WT or Hx−/− mice with SCD (Fig 7B). Therefore, Hp is required for the association of Hb with HDL and for the proinflammatory role of HDL.

### The effect of D-4F on inhibition of p-HDL and its mechanism

**Fig 8:**
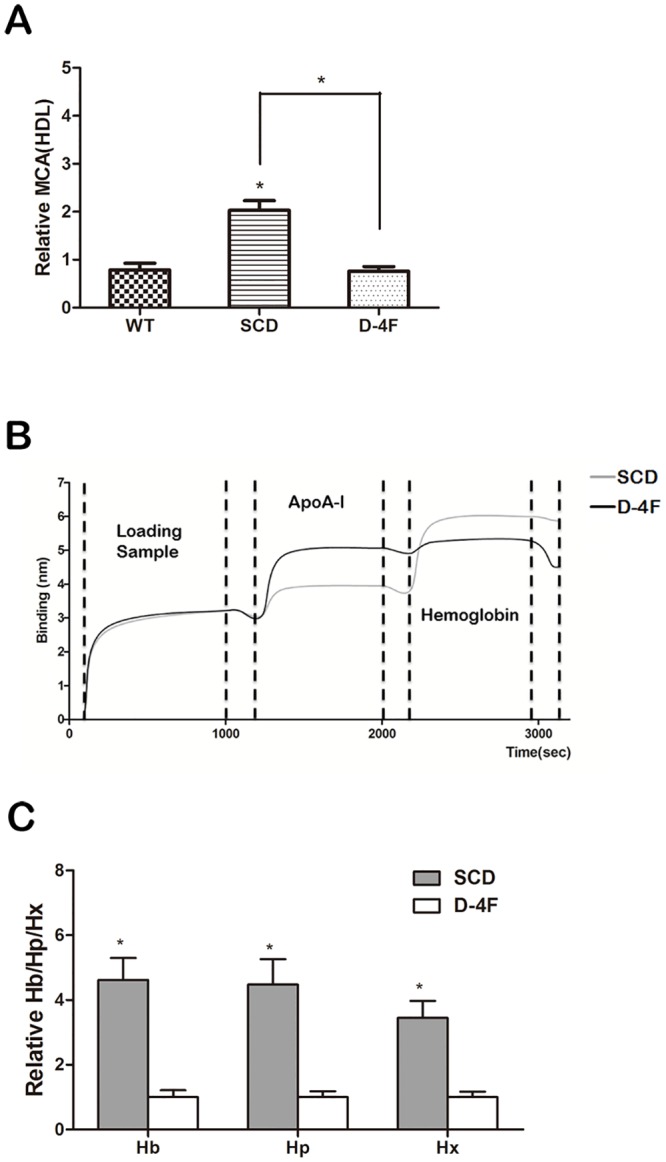
D-4F inhibits the proinflammatory properties of HDL and increases the Hb isolated from HDL. Three groups of mice (n = 8 each) were compared: control, mice with SCD and mice with SCD that were treated with D-4F (10μg/day for 4 weeks) (A) D-4F, an ApoA-1 mimetic peptide, increased the anti-inflammatory properties of HDL (HII<1). (B) Results of analysis with the Octet system to detect interactions among proteins. Biotin-labeled HDL from SCD mice treated with D-4F or placebo was loaded and detected with anti-ApoA-1 and anti-hemoglobin antibodies. D-4F decreased the Hb level in HDL by making Hb dissociate from HDL. (C) Levels of hemoglobin (Hb), haptoglobin (Hp) and hemopexin (Hx) associated with HDL were reduced by the administration of D-4F (* = p<0.005).

To further determine whether the proinflammatory properties of HDL are promoted by the association between Hb/Hp/Hx complexes and ApoA-1 during the conversion process of normal anti-inflammatory HDL into p-HDL, we attempted to understand the effect of D-4F, an ApoA-I mimetic peptide, on Hb/Hp/Hx complexes. In this study, we found that the proinflammatory role of HDL was converted into an anti-inflammatory effect by injection of D-4F in SCD mice (Fig 8A). In addition, we found that less Hb was bound with HDL in the D-4F-treated mice. Therefore D-4F decreased the associated Hb in HDL. Compared with the control group (placebo), D-4F decreased Hb, Hp and Hx associated with HDL in SCD mice (Fig 8B and 8C). Accordingly, D-4F, a mimetic peptide of ApoA-1, can restore the anti-inflammatory properties of HDL through a mechanism involving Hb/Hp/Hx complexes.

## Discussion

In our previous study, cell-free Hb was increased in the SCD group because large mounts of Hb could be released into the plasma for hemolysis. Hb was not only a marker of SCD, but also played an essential role in vascular remodeling in SCD. Hb holds a close relationship with p-HDL, as its accumulation promotes the proinflammatory effect of HDL. In our previous study, Cell-free Hb was increased in the SCD group because large amounts of Hb could be released into the plasma for hemolysis. As stated above, we have achieved our goal for this study of finding the mechanism underlying the association of Hb with p-HDL. Large quantities of Hb can be released into the circulation during hemolysis causing a very high cell-free Hb level in plasma, which lead to the change of inflammatory mircoenvironment and subsequent accumulation of inflammatory factors [22].

It used to be considered that HDL totally played anti-inflammatory and anti-oxidation roles with its protection on vascular pathology [23, 24]. However, HDL can be converted into being proinflammatory with only fewer anti-inflammatory particles when the body is affected by chronic oxidative stress [25]. This change in role of HDL is caused by loss of its anti-inflammatory properties. In the meantime, the accumulation of inflammatory molecules can induce the proinflammatory properties of the HDL cargo, which promote the LDL-induced inflammatory response, higher levels of MCP-1 and monocyte chemotaxis [7, 24, 26]. ApoA-1, PON1, lecithin-cholesterol acyltransferase (LCAT), and other anti-inflammatory factors can be disasscoiated from HDL, but the inflammatory factors take the site to bind with HDL instead [25].

In our study, we demonstrated that in SCD the process of Hb/Hp/Hx binding with HDL was achieved by the association firstly between Hb and Hp (Fig 6). Associated Hb can impair the anti-inflammatory function of HDL in health. ApoA-1 was required by Hp–Hb complexes, and ApoA-1 is the main lipoprotein of HDL, with its function of reverse cholesterol transport [27–29]. Hb, Hp, and Hx can be measured in ApoA-1 particles (Fig 3) and also associate with ApoA-1 in HDL (Fig 4). We found that the association of Hb/Hp/Hx complexes with HDL was increased in SCD patients and animal models, but not in controls (Table 3, Figs 3 and 4). Furthermore, HDL associated with Hb/Hp/Hx complexes could be converted into p-HDL.

In SCD, Hb could associate with ApoA-1 in HDL to make HDL change into p-HDL. But Hb was not elevated in SCD mice with Hp−/− (Fig 5) showing that Hb was disassociated effectively from HDL in spite of hemolysis. Moreover, there was decreased p-HDL if Hb was removed from HDL in Hp-/- SCD mice (Fig 7). Indeed, it has been shown that Hb was removed by monocyte–macrophages via CD163 or endocytosed by hepatic cells to induce the non-inflammatory clearance [16, 30]. Without the association with Hp, Hb can lead much more serious oxidative damage to tissues and organs during hemolysis [31]. Therefore, Hp is considered to be effective protection from the effects of hemolysis. A large quantity of released Hb and the consequent inflammatory response can be seen to be the basis of SCD, with these higher levels of Hp acting as the bridge to connect Hb with HDL. Therefore, our data in this study suggest the large amounts of released Hb are one of the main mechanisms for the proinflammatory effect of HDL.

D-4F is an ApoA-1 mimetic peptide, which was has been shown to have anti-inflammatory properties [32]. Previously, our laboratory demonstrated that D-4F could prevent HDL from becoming proinflammatory and return it to its anti-inflammatory state [25]. The results of this research show that D-4F can make p-HDL regain its anti-inflammatory action. The mechanism involves D-4F decreasing the Hb/Hp/Hx levels associated with HDL. Lower levels of Hp and Hx have a close relationship with the inhibition of inflammation and can influence the binding of Hb to HDL so that there is less Hb associated with HDL. The anti-inflammatory properties of D-4F are connected to reduced linoleic acid and arachidonic acid, and D-4F promotes HDL-mediated cholesterol metabolism. The reverse cholesterol transport can clear out proinflammatory lipids to reduce the inflammatory response in the body.

In summary, the data in our study suggest that Hb/Hp/Hx complexes have the ability to combine with HDL in SCD patients or animal models. Under inflammatory conditions, Hb/Hp/Hx complexes promote the proinflammatory properties of HDL. Pro-inflammation is the main side of HDL in SCD, and Hp is the critical protein controlling the association of Hb with p-HDL as well as changes in the inflammatory properties of HDL. On this basis, we have found a close relationship between the production of HDL and the inflammatory response in the body, which can be tested by hs-CRP and other markers in the clinic. We have also demonstrated that the presence of p-HDL was due to the content of Hb and ApoA-1. The ApoA-1 mimetic peptides (D-4F) can make p-HDL recover to be anti-inflammatory by decreasing the levels of Hb bound to the HDL.

As our previous study, p-HDL cannot perform the protection from inflammatory vascular damage, but induced this inflammatory response. Furthermore, the thickness of vessel wall can be lightened with improvement on its function by decreasing the released inflammatory factors and the production of p-HDL [19]. p-HDL may be more important than the level of HDL-c during the development of the inflammatory response. Therefore, it is reasonable to study the changed protein components in p-HDL to establish a marker for early diagnosis in related diseases and the effective administration of preventive treatments. Accordingly, our laboratory hopes to do further research on the mechanism on the production of p-HDL and the treatment of D-4F. It may be also a good and promising target to study inflammatory damage of vessels and the related mechanism.

## Supporting Information

S1 Fig Plasma hemoglobin (Hb), haptoglobin (Hp) and hemopexin (Hx) in HDL are not linearly correlated with lipid hydroperoxide (LOOH) and HDL inflammatory index (HII) in control group. Results in healthy donors (n = 28): ApoA-1, Hb, Hp and Hx levels in plasma (A1–A4, respectively) and those associated with ApoA-1 (B1–B4, respectively) vs.LOOH content in ApoA-1. ApoA-1, Hb, Hp and Hx levels in plasma (C1–C4, respectively) or those associated with ApoA-1 (D1–D4, respectively) vs.HII. Linear regression was performed individually and p values and R2 values are shown in the figures. (NS = not significant)
(TIF)

S2 Fig All the raw data of Fig 1 and S1 Fig. The data of X-axis (left column) and Y-axis (right column) in each picture were shown separately. Linear regression was performed with these data. ApoA-1, Hb, Hp and Hx levels in plasma (A1–-A4, respectively) or ApoA-1 particles, Hb, Hp, and Hx associated with ApoA-1 (B1–B4, respectively) vs. LOOH content in ApoA-1 particles. ApoA-1, Hb, Hp and Hx levels in plasma (C1–C4) or those associated with ApoA-1 (D1–D4) vs. HII measured by monocyte chemotaxis activity (MCA).
(TIF)

S3 Fig All the raw data of Fig 2. It shown the raw data in each picture, including X-axis (left column) and Y-axis (right column) in each group. Linear regression was performed individually. Hb levels in the plasma (A1–A4 and C1–C4). Relative Hb [ApoA-1] = Hb associated with ApoA-1 particles from HDL (B1–B4 and D1–D4). Fig A1-A4 and B1-B4 present the data from the SCD group (n = 32) and Fig C1-C4 and D1-D4 are those from healthy donors (n = 28).
(TIF)

S4 Fig **(A) Original result of** Fig 4A.This file shown the original pictures of Fig 4A (Immunoblot) and the experiments were repeated to demonstrate its truth. The first one was chosen for publication. SCD-HDL = HDL sample of SCD group; WT-HDL = HDL sample of wild-type (WT) group; SCD = plasma of SCD group; WT = plasma of WT group. Plasma standard was used for positive control. **(B) Original result of** Fig 4C. It shown all the original result of Fig 4C (Immunoblot), which shown the levels of hemoglobin (Hb), haptoglobin (Hp) and hemopexin (Hx) associated with ApoA-1 particles. In each group (SCD/WT), all bands in the same line were from different mice. The statistical calculation for grey level can be performed based on all the 4 bands of Hb/Hp/Hx in each group. The right part of this figure was used for publication.
(TIF)
