# Supplementary figures and images for: The Mechanism of Proinflammatory HDL Generation in Sickle Cell Disease Is Linked to Cell-Free Hemoglobin via Haptoglobin
Source: PLoS One. 2016 Oct 7;11(10):e0164264. doi: 10.1371/journal.pone.0164264 (PMC5055316; doi:10.1371/journal.pone.0164264)

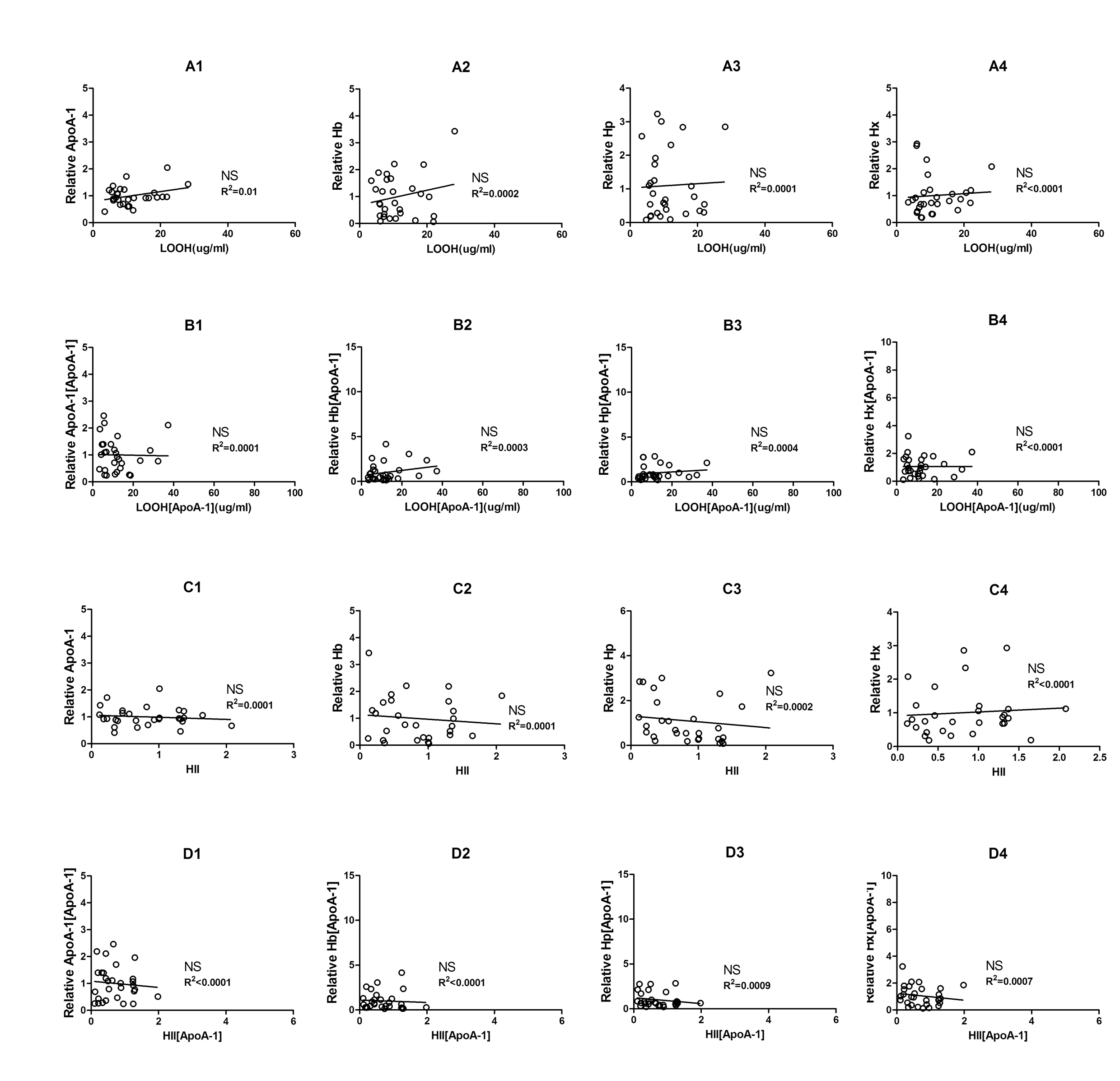

Supplement: S1 Fig — Results in healthy donors (n = 28): ApoA-1, Hb, Hp and Hx levels in plasma (A1–A4, respectively) and those associated with ApoA-1 (B1–B4, respectively) vs.LOOH content in ApoA-1. ApoA-1, Hb, Hp and Hx levels in plasma (C1–C4, respectively) or those associated with ApoA-1 (D1–D4, respectively) vs.HII. Linear regression was performed individually and p values and R2 values are shown in the figures. (NS = not significant) (TIF) [file pone.0164264.s001.tif]

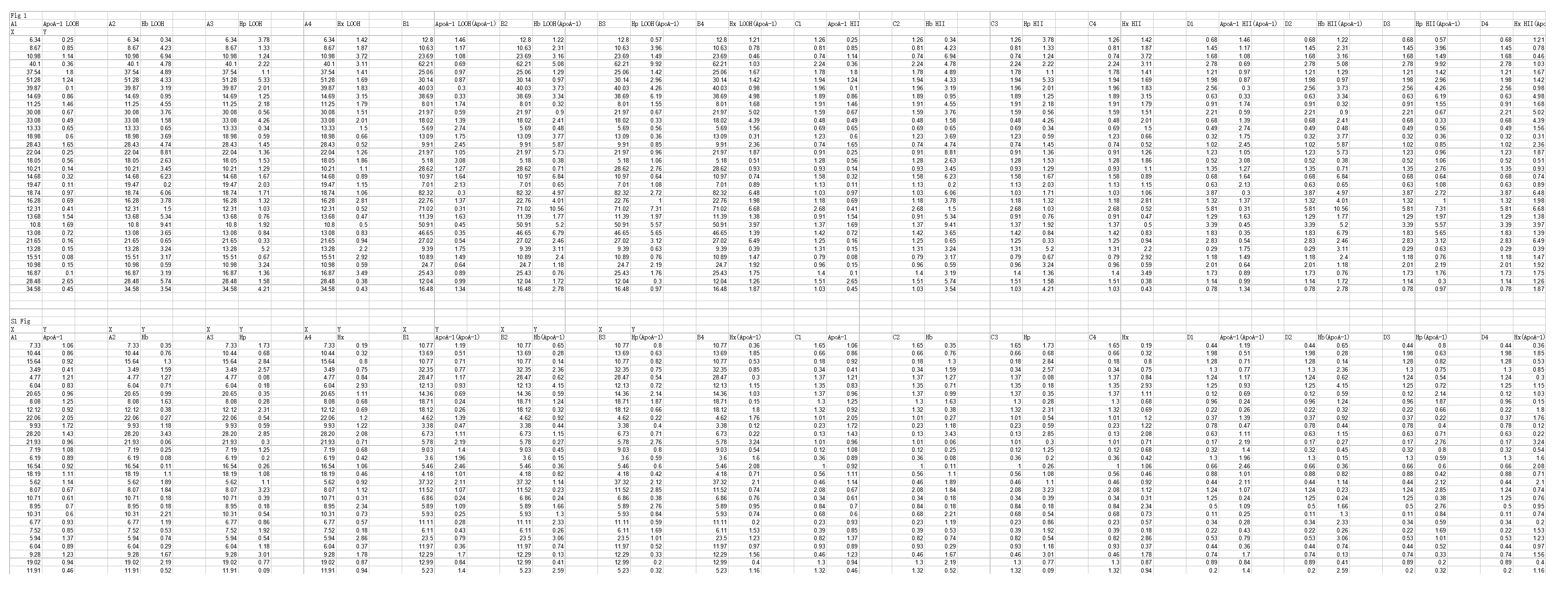

Supplement: S2 Fig — The data of X-axis (left column) and Y-axis (right column) in each picture were shown separately. Linear regression was performed with these data. ApoA-1, Hb, Hp and Hx levels in plasma (A1–-A4, respectively) or ApoA-1 particles, Hb, Hp, and Hx associated with ApoA-1 (B1–B4, respectively) vs. LOOH content in ApoA-1 particles. ApoA-1, Hb, Hp and Hx levels in plasma (C1–C4) or those associated with ApoA-1 (D1–D4) vs. HII measured by monocyte chemotaxis activity (MCA). (TIF) [file pone.0164264.s002.tif]

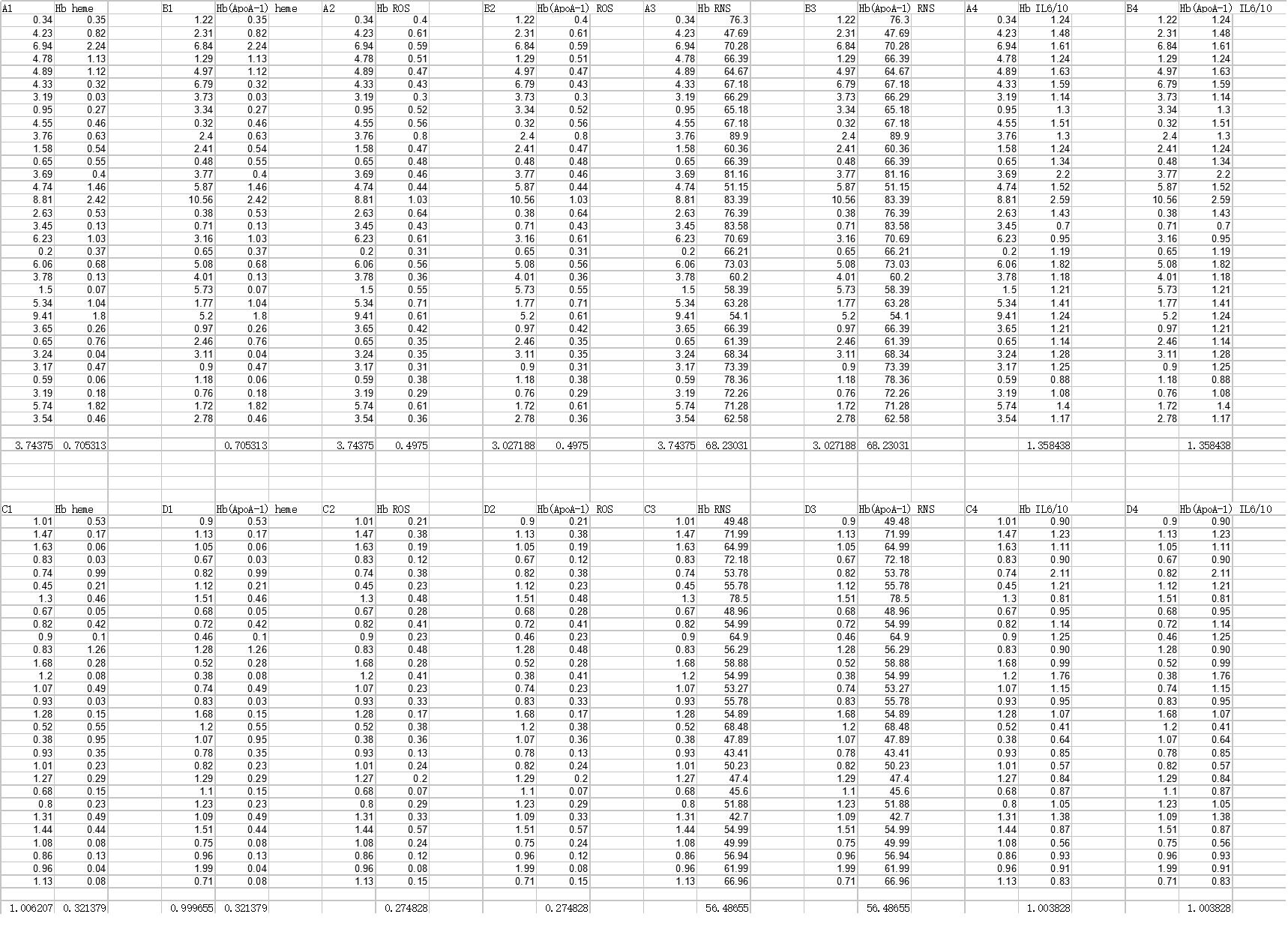

Supplement: S3 Fig — It shown the raw data in each picture, including X-axis (left column) and Y-axis (right column) in each group. Linear regression was performed individually. Hb levels in the plasma (A1–A4 and C1–C4). Relative Hb [ApoA-1] = Hb associated with ApoA-1 particles from HDL (B1–B4 and D1–D4). Fig A1-A4 and B1-B4 present the data from the SCD group (n = 32) and Fig C1-C4 and D1-D4 are those from healthy donors (n = 28). (TIF) [file pone.0164264.s003.tif]

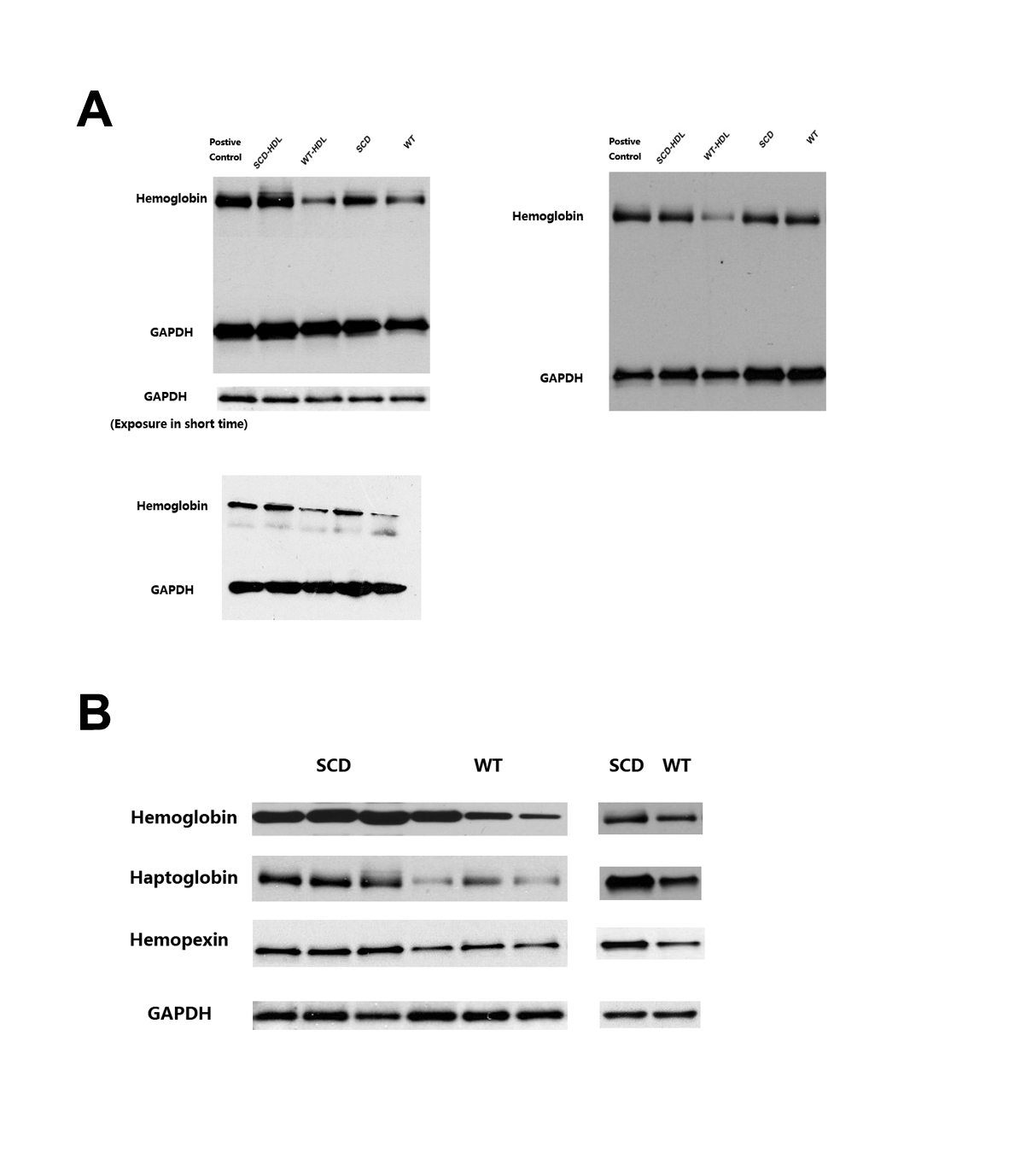

Supplement: S4 Fig — (A) Original result of Fig 4A.This file shown the original pictures of Fig 4A (Immunoblot) and the experiments were repeated to demonstrate its truth. The first one was chosen for publication. SCD-HDL = HDL sample of SCD group; WT-HDL = HDL sample of wild-type (WT) group; SCD = plasma of SCD group; WT = plasma of WT group. Plasma standard was used for positive control. (B) Original result of Fig 4C. It shown all the original result of Fig 4C (Immunoblot), which shown the levels of hemoglobin (Hb), haptoglobin (Hp) and hemopexin (Hx) associated with ApoA-1 particles. In each group (SCD/WT), all bands in the same line were from different mice. The statistical calculation for grey level can be performed based on all the 4 bands of Hb/Hp/Hx in each group. The right part of this figure was used for publication. (TIF) [file pone.0164264.s004.tif]
